# One-Step Fabrication of a Functionally Integrated Device Based on Polydimethylsiloxane-Coated SiO_2_ NPs for Efficient and Continuous Oil Absorption

**DOI:** 10.3390/ma14205998

**Published:** 2021-10-12

**Authors:** Guannan Ju, Lei Zhou, Chang Jiao, Jiafeng Shen, Yihao Luan, Xinyu Zhao

**Affiliations:** 1 School of Materials Science and Engineering, Shandong University of Technology, Zibo 255000, China; yshxq12138@163.com; 2 China National Accreditation Service for Conformity Assessment, Beijing 100062, China; jiaochang@cnas.org.cn; 3 School of Materials Science and Engineering, Beijing University of Chemical Technology, Beijing 100029, China; shenjiafeng2007@163.com

**Keywords:** functionally integrated device, oil-water separation, superhydrophobic, superoleophilic

## Abstract

The construction of superhydrophobic surfaces necessitates the rational design of topographic surface structure and the reduction of surface energy. To date, the reported strategies are usually complex with multi-steps and costly. Thus, the simultaneous achievement of the two indispensable factors is highly desired, yet rather challenging. Herein, we develop a novel structure engineering strategy of realizing the fabrication of a functionally integrated device (FID) with a superhydrophobic surface via a one-step spraying method. Specifically, silica nanoparticles are used to control the surface roughness of the device, while polydimethylsiloxane is employed as the hydrophobic coating. Benefitting from the adopted superhydrophobicity, the as-fabricated FID exhibits a continuous, excellent oil-water separating performance (e.g., 92.5% separating efficiency) when coupled with a peristaltic pump. Notably, a smart design of incorporating a gas switch is adopted in this device, thereby effectively preventing water from entering the FID, realizing thorough oil collection, and avoiding secondary pollution. This work opens up an avenue for the design and development of the FID, accessible for rapid preparation and large-scale practical application.

## 1. Introduction

Oil spills occurring occasionally during oil exploration and transportation pose great threats to the marine ecosystem, which would cause catastrophe for ocean lives by poisoning animals/plants, damaging their habitat and slowing the reproductive rate [1,2]. The oily water also endangers human health in many ways, such as contaminated sea food and polluted freshwater resources. In this regard, oil-water separation techniques are in great demand for oil spill clean-up. Among them, the functionally integrated device (FID) that enables simultaneous oil absorption and separation has attracted intensive attention due to its merits of cost-efficiency, energy-saving, and environmental benignity [3,4]. A Functionally integrated device (FID) refers to a miniaturized system with specific function and structural design in one device, which can function in a sequential manner to complete designated missions. The reported FID is mainly focused on the miniaturized devices with a sealed or fully open design [5,6]. These devices have been demonstrated for applications in advanced research fields or for practical uses, including biomimicking devices, macroscopic supramolecular assembly, mini-generators, active oil absorption and separation, etc. [7,8]. Besides, most reported FIDs are only effective to light oils with low viscosity, yet do not work well for viscous heavy oil. The strong demand for absorbing heavy oil with high viscosity drives researchers to further improve the structure and functions of the FIDs. To enhance the absorption/separating efficiency of the FID, it is necessary for constructing superhydrophobic/superoleophilic surfaces realizing the thorough oil separation from oily water [9,10,11,12,13,14,15,16,17,18,19,20]. Typically, superhydrophobic surfaces can be achieved by surface modification and construction of topographic surface structure [21,22]. For example, a variety of top-down approaches (e.g., lithography [23], laser beam treatment [24]) and bottom-up approaches (e.g., self-assembly [25], 3D printing [26]) have been developed to construct micro- and nanostructures on surfaces. In addition, the adoption of coatings with low surface free energy is a popular means for surface modification and enhancement of hydrophobicity. Despite research progress, the current fabrication methods are limited due to the involvement of multiple step treatments and noble-metal raw materials (e.g., Au and Ag), making them inaccessible for rapid preparation (i.e., within several minutes) and large-scale practical application [27,28,29]. Therefore, the simultaneous achievement of the rationally designed surface structure and reduced surface energy are highly required, yet rather challenging.

**Figure 1:**
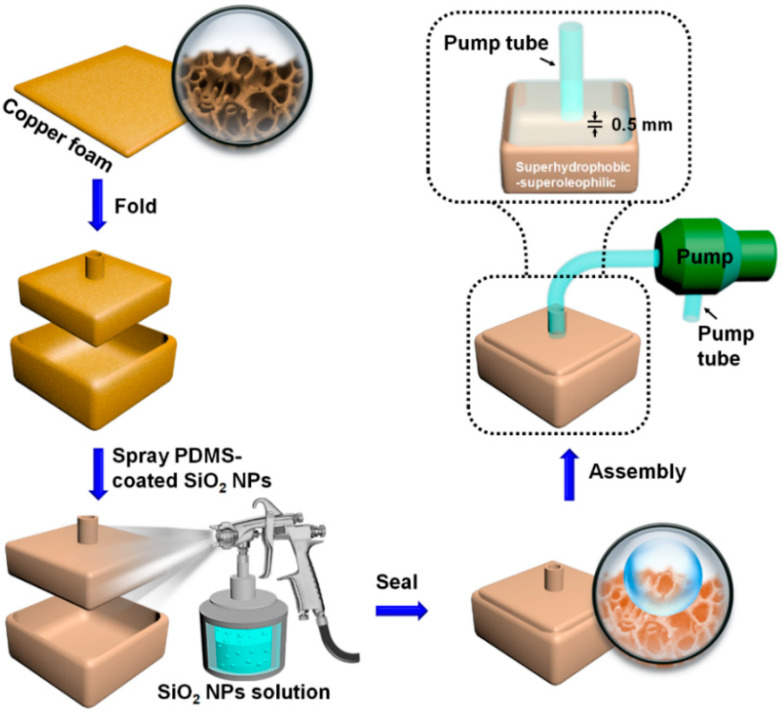
Schematic Illustration of the one-step spraying strategy to fabrication of the FID based on the SiO_2_@PDMS composite and the assembly of the as-prepared FID into an oil-water separating apparatus.

In this article, we demonstrate a facile, cost-effective spraying strategy for fabricating a superhydrophobic FID for light oil absorption using the polydimethylsiloxane-coated silica nanoparticles (SiO_2_@PDMS) composite supported by a copper foam substrate (Figure 1). Specially, the SiO_2_ NPs with high thermal stability and good mechanical strength are used to control the surface roughness of the device [30], while the PDMS with hydrophobic property, high chemical stability and good weatherability is employed as the low-surface-energy coating [31,32]. When connected to a peristaltic pump using a tube, the FID performs as a collector, realizing continuous and thorough oil collection from water. The oil-water separation ability of our FID under some simulated real conditions of use (i.e., salt water, droplet oil on the water, and oil underwater) as well as the oil-water separation recycling tests of our FID were evaluated to ascertain its performance stability under practical application environment.

## 2. Materials and Methods

### 2.1. Materials and Instruments

All chemicals in this study were used as received: copper foam was obtained from Anping Xinlong Wire Mesh Manufacture Co., Ltd., Anping, China; Press varnish, ethanol, xylene, n-hexane, n-heptane, dichloromethane, were purchased from Sinopharm Chemical Reagent Beijing Co., Ltd., Beijing, China. PDMS was obtained from Dow Corning (pre-polymer, Sylgard 184 silicone elastomer, fluid viscosity 5500 cps). SiO_2_ NPs were obtained from Anhui Evolution Silicon Nano Material Technology Co., Ltd., Anhui, China. Deionized water was used for all the tests and experiments. All chemicals were used as received without further purification.

Scanning electron microscope (SEM) images and energy dispersive spectroscopy (EDS) images were obtained with a Zeiss Supra55, Zeiss, Oberkochen, Germany at 20.0 kV. Transmission electron microscope (TEM) images were obtained with a Tecnai G2 F20 S-TWIN, Thermo Fisher Scientific, Waltham, MA, USA. Contact angle (CA) values were measured on an OCA20 instrument (DataPhysics Instruments GmbH, Filderstadt, Germany) using a water droplet of 4 μL volume, from Data Physics Instruments Gmbh, Filderstadt, Germany. The. Photographic images and movies were taken with a Sony camera (A6000), Sony, Tokyo, Japan. The spray gun was purchased from Shanghai Six Kam Electromechanical Equipment Co., Ltd., Shanghai, China (product NO. W-71, nozzle orifice ϕ = 1.5 mm). The peristaltic pump (BT100S-1) was purchased from Baoding Rongbai Precision Pump Co., Ltd., Hebei, China.

### 2.2. Fabrication of the Oil-Water Separating Apparatus

The preparing process of the FID was described below. First, two pieces of copper foam with a designed dimension of 4.5 × 4.5 cm^2^ and 4.3 × 4.3 cm^2^ were manually folded into two boxes without cover (2.5 × 2.5 × 1 cm^3^ and 2.3 × 2.3 × 1 cm^3^). Meanwhile, the top of the smaller copper foam box was punched a hole (diameter: 5 mm) following the schematic procedure in the Supplementary Figure S1. The copper foam box was cleaned under ultrasonication in ethanol and subsequent deionized water for three times each, followed by a drying process in an oven at 60 °C. Second, the PDMS precursor was prepared by mixing the PDMS base and the curing agent in a 10:1 weight ratio. Then the PDMS precursor, SiO_2_ NPs, and heptane solvent were mixed together in a dilution ratio 1 (i.e., 0.3 g SiO_2_ NPs: 0.2 g PDMS: 20 mL heptane) [33,34,35], before an ultrasonic treatment for 15 min was conducted to form a suspension. The dilution ratio was 0.5, 1.5, 2, and 2.5 when 10, 30, 40, and 50 mL heptane was added with the same amount of SiO_2_ NPs and the PDMS precursor (Figures S2 and S3). Third, the suspension was sprayed onto the surface of the copper foam or other substrates using a spray gun with a pressure of 0.3 MPa which was placed 15 cm above the substrate and waved back and forth for 80 s. After the SiO_2_ NPs dispersed solution was uniformly sprayed on the inner and outer surfaces of the copper foam boxes, the sample was dried in an oven for 60 min at 60 °C to obtain the superhydrophobic surface. Two copper foam boxes had finished surface modification were assembled together to form the superhydrophobic FID. Finally, the as-prepared FID was combined with a peristaltic pump to form a continuous oil-water separating apparatus. Notably, a 0.5 mm air gap is intentionally set between the tube and the bottom of the FID, serving as a smart gas switch to regulate the absorption, separation and collection of the oil.

### 2.3. Test of Superhydrophobicity/Superoleophilicity

In order to distinguish n-heptane from water, we dyed the n-heptane a red colour with a solvent dye called press varnish and dye the water a blue colour with dark-blue ink. Dyed water or oil droplets were positioned onto the surface of the as-prepared FID using a plastic dropper. Water contact angle (WCA) and oil contact angle (OCA) measurements on the as-prepared materials were carried out with 4 μL droplets on an OCA 20 instrument (DataPhysics Instruments Gmbh, Filderstadt, Germany).

### 2.4. Continuous and Controllable Oil-Water Separation Process

In order to realize continuous and controllable oil-water separation by using the as-prepared oil-water separating apparatus, we performed the following experiment. First, we dyed n-heptane to red with press varnish to distinguish oil layer from the mixture, and then prepared the oil-water mixture by mixing 80 mL dyed n-heptane and 200 mL water. Second, the device connected with the pump tube was then placed on the oil-water mixture, and the other end of the pump tube was placed into a collecting vessel. The generating pressure of the peristaltic pump was about 0.1 MPa and the revolution speed of peristaltic pump was 60 rpm. The dyed-red oil could be rapidly absorbed upon contacting the device and further pumped away from the interior of the device by starting the peristaltic pump. When most of the oil was pumped out from the system, air was continuously pumped into the pump tube instead of oil to terminate the oil collection and avoid the secondary pollution. This process was a continuous oil-water separation and collection process. Third, in order to restart the oil-water separation and collection, the extra oil (10 mL or more) was added into the system again. Meanwhile, the oil would replace the air in the pump tube and would be collected to the vessel by starting the pump again, thus allowing a continuous and controllable working mode.

## 3. Results

### 3.1. Surface Morphology and Wetting Behavior of the FID

**Figure 2:**
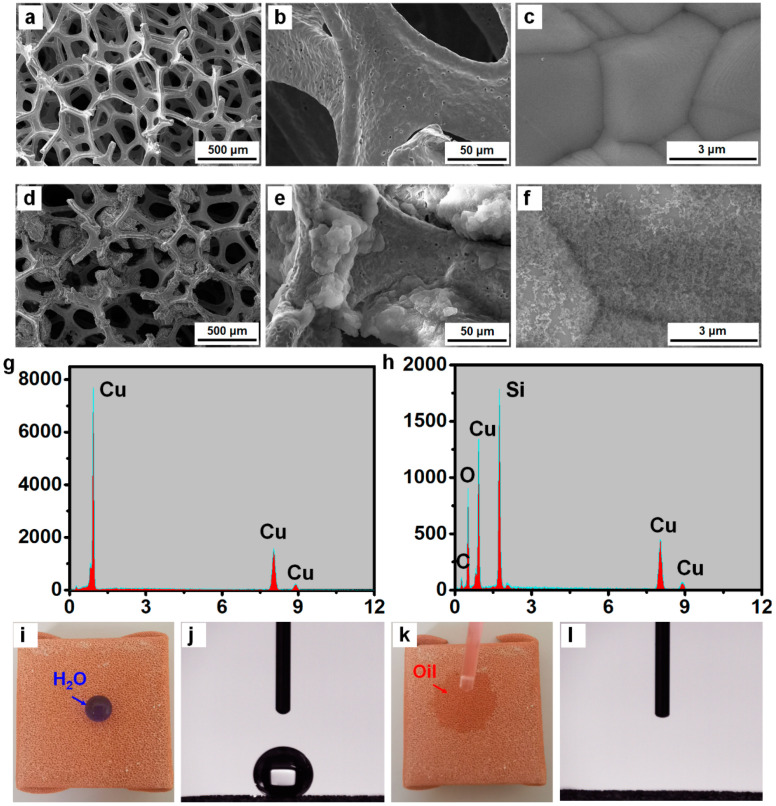
SEM images of the copper foam (**a**–**c**) before and (**d**–**f**) after surface modification using the SiO_2_@PDMS composite. EDS images of the copper foam (**g**) before and (**h**) after surface modification using the SiO_2_@PDMS composite. (**i**,**j**) Photograph of the water droplet dyed blue placed on the FID and the corresponding WCA. (**k**,**l**) Photograph of the oil droplet dyed red placed on the FID and the corresponding OCA.

The nano- and micro-meter scale structures in conjunction with a low-surface-energy coating are indispensable for preparing superhydrophobic surfaces. To simultaneously achieve the optimal topographic structure and the low-surface-energy coating by directly spraying the SiO_2_@PDMS composite, the effects of the ratio of PDMS, dilution ratio and spraying time on WCA are investigated (Figure S4). In order to check the surface roughness, we characterized the surface morphology of the copper foam before and after the spraying process. Before the spraying step, the scanning electron microscopy (SEM) image demonstrated that the pristine copper foam possesses ample staggered holes with an averaged diameter of ~500 μm, as shown in Figure 2a. From the magnified SEM image (Figure 2b,c), it is observed the surface of skeleton is smooth, which is comprised of Cu grains with the size of ~3 μm. After surface modification using the SiO_2_@PDMS composite, the porous structure of the copper foam is still maintained (Figure 2d), which ensures that the oil could easily penetrate into the pores under the capillary force. The magnified SEM images (Figure 2e,f and Figure S5) show that a layer of SiO_2_@PDMS composite with close-packed, nano- and microscale structures is coated on the porous foam skeleton. Such micro-nanostructures can promote the substrate from hydrophobic state to superhydrophobic state. Simultaneously, the diluent PDMS act as a low-surface-energy coating for the realization of superhydrophobicity, thus avoiding extra surface modification steps as required in most reports. The roughness data before and after coating copper foam with SiO_2_@PDMS composite coating is provided by employing the Brunauer-Emmett-Teller (BET) surface area analysis. The specific surface area of the copper foam increases from 8.21 to 20.08 m^2^ g^−1^ after SiO_2_@PDMS coating modification, which is favorable for the construction of the superhydrophobic coating and the rapid oil absorption by providing the sufficient contact between the nanostructured surface and the oil. In addition, the surface composition before and after the surface modification using the SiO_2_@PDMS composite are characterized by the energy dispersive spectrometer (EDS). The bare copper foam presents a relatively smooth surface with the major element of cuprum in the selected region (Figure 2g). While the strong absorption peaks of the silicon, oxygen, and carbon elements imply the presence of the SiO_2_@PDMS composite coating (Figure 2h). In this way, the fast and simple spraying method ideally realizes the two essential factors to obtain superhydrophobicity.

To prove the superhydrophobicity of the as-prepared FID, the wettability of the as-prepared FID is evaluated with observable liquid tests and water contact angle (WCA) measurements. As is shown in Figure 2i,j, when a drop of dyed-blue water droplet is dropped onto the surface of the FID, the water droplet (the droplet volume is 4 μL) easily stands on the surface to form a liquid ball giving a WCA of ~150° due to its relatively high surface energy of 72 mN∙m^−1^. The water contacting situation can be interpreted by the Cassie-Baxter model, which assumes that water cannot totally fill the grooves of the rough surface, therefore leaving space for trapping air [21,22]. In contrast, the oil (n-heptane) droplet rapidly spreads out on the surface of the copper foam giving an oil contact angle (OCA) of ~0°, which is because that the surface energy of the used oil (22.1 mN∙m^−1^) is comparable with that of the SiO_2_@PDMS composite modified coating (Figure 2k,l). The dyed-red n-heptane penetrates the modified surface when an excessive amount of it is applied. While the blue water was thoroughly blocked within the device because of its superhydrophobicity, leading to complete separation of the oil-water mixture (Supplementary Figure S6 and Video S1). To evaluate the performance of our FID under practical application conditions (e.g., spilled oil in saline water), an open device with superhydrophobic/superoleophilic property was used for absorbing the oil droplets floating on the surface of water (Figure S7). The as-prepared device completely absorbs the floating oil droplets through manual movements. While the FID was coated with superhydrophilic/underwater-superoleophobic coatings, the water could pass through easily while oil droplet was blocked in the device. Therefore, from the point of flux, superhydrophilic/underwater-superoleophobic device could be effective for treating dilute oil-water solution. The superhydrophobicity of our FID is also proved through the change in WCA value of copper foam substrate before and after coating PDMS and SiO_2_@PDMS (Figure S8). The WCA increases from 92.5 to 137.8° after coating PDMS and to 150.2° after coating SiO_2_@PDMS, clearly showing the superhydrophobicity of our FID. Meanwhile, the as-prepared FID could withstand weak acidic or alkalescent environmental conditions. Aqueous solutions with different pH value (pH = 2–13) have WCA of ~151.5° on the treated surfaces, as shown in Figure S9. These remarkable wettability differences suggest that the FID would provide a feasibility for the subsequent oil absorption and collection.

### 3.2. The Versatility of this Spraying Method

**Figure 3:**
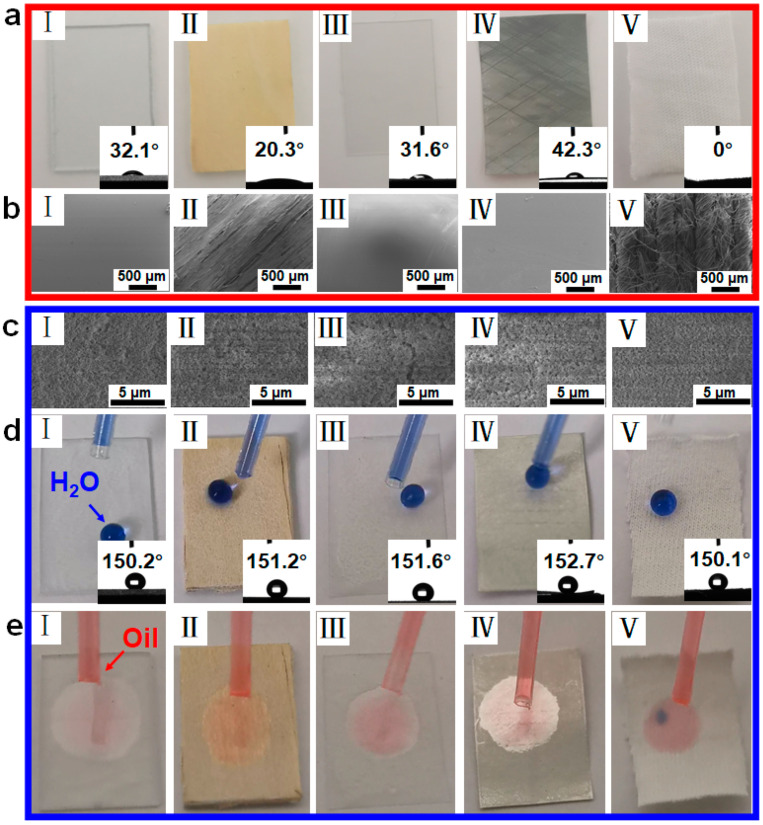
(**a**) SEM images and (**b**) photographs of (I) glass sheet, (II) wood sheet, (III) transparent polypropylene sheet, (IV) aluminium flake, (V) thin cotton with the same size of 25 × 20 mm^2^. (**c**–**e**) SEM images and photographs of the modified surfaces with superhydrophobicity and superoleophilicity.

Using the porous copper foam as a model substrate, we have demonstrated the feasibility of the spraying method to obtain a superhydrophobic/superoleophilic surface. However, copper foam is not ready-made in many situations of normal life. To clarify the versatility of this method, we wondered whether materials available in daily life were suitable to be used as the substrates. The substrate versatility of our one-step spraying method is evaluated by constructing a superhydrophobic surface on different substrates, such as (I) glass sheet, (II) wood sheet, (III) transparent polypropylene sheet, (IV) aluminium flake; (V) thin cotton (Figure 3a). The SEM images in Figure 3b,c show the surface morphology of different substrates before and after surface modification using the one-step spraying method. Raw substrates show a typical smooth surface. However, the modified substrates show rough morphologies due to the random aggregated of SiO_2_ NPs in the PDMS coating. The wetting behaviors of the as-prepared superhydrophobic surface on different substrates are checked by observable liquid tests and WCA measurements. The WCA values of different raw substrates are all less than 50.0°, showing their intrinsic hydrophilic properties (inset of Figure 3a). After modification with our strategy, all of these substrates successfully become superhydrophobic with the WCA values larger than 150.0° (Figure 3d). While the water droplets easily stand on all the modified surfaces and slide off the gradient surface (Figure 3d). When dipping the dyed-red oil droplet on the modified surfaces, it spreads rapidly on the whole surface and is completely absorbed within 1 s (Figure 3e), which shows the superhydrophobicity/superoleophilicity of the as-prepared substrates. These results indicate that the one-step spraying strategy is universal for the different types of substrates, which has been a significant research topic for the oil-water separation application.

### 3.3. Continuous and Controllable Oil-Water Separation and Collection

**Figure 4:**
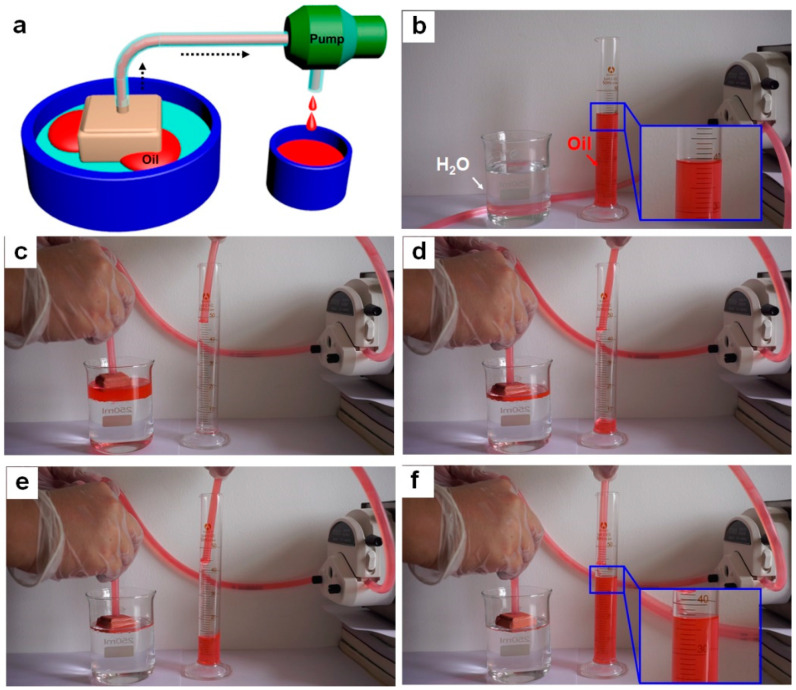
(**a**) Schematic illustration of the oil-water separating apparatus. (**b**–**f**) Stepwise snapshots in the continuous oil-water separation and collection process.

To simulate the treatment of oil spills on the sea, an oil-water separating apparatus is set up using the as-prepared FID and a peristaltic pump to rapidly absorb and collect the oil from the water surface, as shown in Figure 4a. The dyed-red n-heptane (40 mL) is placed into a beaker filled with water (150 mL) to form an oil-water mixture, and the as-prepared FID is subsequently placed on the surface of oil-water mixture. The continuous separation and collection processes are realized via the FID with a smart design of the gas switch and a peristaltic pump. Upon starting up the peristaltic pump, the dyed n-heptane is rapidly absorbed into interior of the FID as a result of the superhydrophobicity and superoleophilicity, and subsequently gathered into the volumetric cylinder through the pump tube (Figure 4b–d). With the proceeding of pumping, most of n-heptane around the FID is absorbed continuously into the interior of the FID, while keeping the water blocked outside (Figure 4e). Finally, about 37 mL of the red n-heptane is collected into the volumetric cylinder as the running time increases (Figure 4f). The oil-water separating efficiency is calculated to be 92.5% by comparing the collected oil volume and the original oil volume except residual oil in the pump tube. This continuous oil-water separation process can be recycled more than three times. Subsequently, the residual oil in the pump tube is poured into the volumetric cylinder, leading to the collected volume of 39.2 mL and obtaining a separating efficiency to be 98% (Figure S10). Moreover, the efficient oil-water separation and oil collection have been achieved in salt water/oil mixture system, showing that our FID has stable superhydrophobicity in weak saline environment (Figure S11 and Video S2). Notably, no water is pumped in the FID during the oil collecting process, successfully avoiding the secondary pollution. The thorough collection of oil without water is attributed to the smart design of the gas switch and the excellent superhydrophobicity of our FID. The superhydrophobic FID wall can hold a layer of air and connect the inner space. When the pressure difference between inside and outside of the FID created by the pump is too large, or there is little oil in the interior of the FID, the air layer could allow air outside enter the FID rather than water, thereby realizing thorough oil collection, and avoiding secondary pollution.

**Figure 5:**
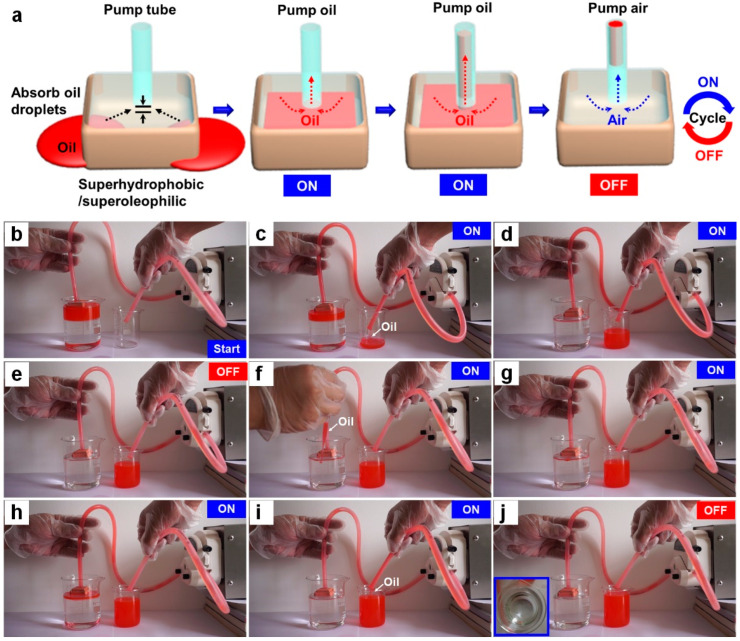
(**a**) Schematic for the oil-water separation mechanism based on a smart design of gas switch. (**b**–**j**) Corresponding optical snapshots of collecting oil with starting (ON)—stopping (OFF)—starting again (ON) function.

To further explore the controllable oil-water separation mechanism of our FID, a cyclic adsorption test is performed (Figure 5a). When the oil-water separating apparatus is started, a large amount of the dyed-red n-heptane is absorbed into the interior of the FID, due to its superhydrophobic/superoleophilic properties (Figure 5b–d). At the ON state, the top oil surface in the FID maintains higher than the bottom of the embedded pump tube, so the oil is easily pumped into the collector (i.e., a beaker). When most of dyed-red n-heptane around the FID is pumped out, the top oil surface in the FID decreases until the location of air gap to form a gas switch due to the entrance of air, and this state is indicated as OFF (Figure 5e). The entrance of air rather than the water at the OFF state is attributed to the superhydrophobic surfaces and gas switch of the FID. The oil collecting process can be readily restarted by the addition of extra oil. Obviously, the initiate dyed n-heptane is still absorbed quickly into the interior of the FID. When the height of the oil stored in the interior of the FID reaches the bottom of the pump tube, the n-heptane is re-pumped into the beaker, turning the system into “ON” state again (Figure 5f). The “ON-OFF-ON” state can be cycled many times by adding extra oil, realizing the controllable oil separation and collection (Figure 5g–j and Video S3). These results successfully verify the effectiveness of the gas switch for controllable oil-water separation, leading to thorough oil collection without the secondary pollution.

**Figure 6:**
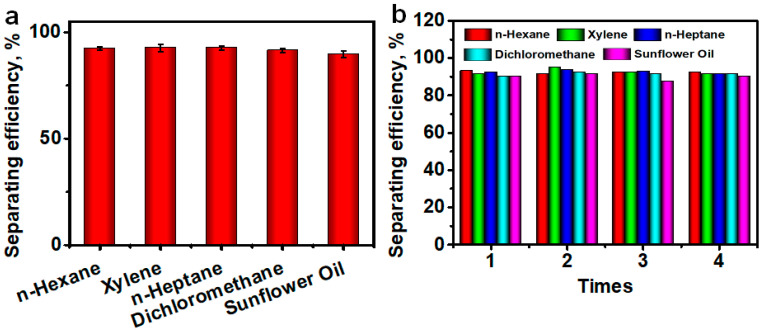
(**a**) Oil-water separating efficiency of five oil types: n-hexane, xylene, n-heptane, dichloromethane and sunflower oil in one round of oil-water separation. (**b**) Recycled number and separating efficiency of different type of oil.

Considering the fact that the spilled oil usually contains various compositions, five kinds of oil-water mixtures (i.e., n-hexane-water, xylene-water, n-heptane-water, dichloromethane-water and sunflower oil-water mixtures) are used to evaluate versatility of our oil-water separation apparatus. Using the as-prepared apparatus for continuous oil-water separation, we carried out oil-water separation tests of the above five mixtures for four cycles and calculated the corresponding separating efficiency. The oil-water separating efficiency for all five kinds of oil with densities of either lower or higher than that of water reach >90% in the first test, as shown in Figure 6a and Figure S12. In the next four cycles, as summarized in Figure 6b, five types of oil were also used following the same procedure. The results demonstrated that the as-prepared FID shows a steady separating efficiency higher than 90% in four identical repeated oil-water separation processes regardless of the oil types, thus indicating that the as-designed oil-water separating apparatus could act as a potential candidate with good durability and reproducibility for the oil-water separation in industrial applications. However, both the separating efficiency and WCA start to decrease after five cycles. This may be because that a small portion of SiO_2_ NPs particles is peeled off from the FID surface after multiple washing-drying-testing cycles due to the relatively weak adhesion of SiO_2_ NPs on the surface (Figure S13).

## 4. Conclusions

In summary, a one-step spraying strategy is successfully demonstrated for constructing a superhydrophobic FID for efficient light oil-water separation. The superhydrophobicility of the fabricated FID is realized by virtue of a delicate surface structure modification by SiO_2_ NPs and a coating of a low-surface-energy surface of PDMS. The evaluating tests prove that our one-step spraying strategy has excellent substrate and oil type versatility. Furthermore, an oil-water separation apparatus with a smart gas switch is set up using the as-prepared FID and a peristaltic pump. Benefitting from the adopted superhydrophobicity and designed gas switch, the apparatus shows controlled oil collection, preventing water from entering the FID, avoiding secondary pollution, and realizing thorough oil collection (i.e., separation efficiency of 92.5%). Although our FID shows excellent performance for light oil absorption, it is limited when absorbing heavy oil with high viscosity. The heavy oil absorption process suspends after a few minutes as the pores of our FID is blocked by the initial adsorbed heavy oil due to its poor fluidity. This work opens up an avenue for the design and development of the FID accessible for rapid preparation and large-scale applications.

## Data Availability

Not applicable.

## Supplementary Materials

The following are available online at https://www.mdpi.com/article/10.3390/ma14205998/s1, Figure S1: Illustration of the folding process of the functionally integrated device. Folding in the dotted line, followed by a simple assembly process ((1)–(5)); Figure S2: TEM images of SiO_2_ NPs with different magnification. Diameter of the as used SiO_2_ NPs is about ~12 nm; Figure S3: Ultrasonic dispersed SiO_2_ NPs solution; Figure S4: The effect of the ratio of PDMS (a), dilution ratio (b) and spraying time (c) on WCA; Figure S5: SEM images with different areas (area 1, area 2, area 3, area 4) and magnification (2000×, 100,000×, 150,000×, 200,000×) for modified copper foam; Figure S6: (a) The as-prepared device and the oil-water mixture. (b) The dyed-red n-heptane could be absorbed immediately and the excessive n-heptane could continuously penetrate the surface. (c,d) While the dyed-blue water would be blocked outside. (e) Schematic diagram of water and oil permeation process; Figure S7: The process for the clean-up of oil droplets suspended or floated in water; Figure S8: Photos of water droplets placed on the copper foam (a) before and (b,c) after the PDMS or PDMS/SiO_2_ NPs modification. Corresponding WCA (d–f), SEM (g–i) and EDS (j–l) of as-prepared surface; Figure S9: Variation of WCA of the as-prepared device under the different pH value; Figure S10: After the residual oil in the pump tube was poured out, the collected oil volume was about 39.2 mL; Figure S11: (a–c) Stepwise snapshots in the continuous oil-water separation and collection process in salt solution. (d) The WCA under different salt concentration; Figure S12: Optical snapshots of the dichloromethane absorbing process under the water surface; Figure S13: Recycled number and separating efficiency of the as-prepared FID in oil-water separation process. Inset: the corresponding WCA of the FID after each cycle; Video S1: The oil-water separation process of the as-prepared device; Video S2: The separation of salt oil-water mixture; Video S3: The continuous and controllable oil-water separation with ON-OFF-ON function. All the videos are accelerated to 3 times.
