# Supplementary material for: One-Step Fabrication of a Functionally Integrated Device Based on Polydimethylsiloxane-Coated SiO2 NPs for Efficient and Continuous Oil Absorption
Source: Materials (Basel). 2021 Oct 12;14(20):5998. doi: 10.3390/ma14205998 (PMC8537208; doi:10.3390/ma14205998)
Supplement: Supplementary file 1 [file materials-14-05998-s001.zip › materials-1403509- SM final.pdf]

materials

Supplementary Material

One-Step Fabrication of a Functionally Integrated Device Based on Polydimethylsiloxane-Coated SiO_2 NPs for Efficient and Continuous Oil Absorption

Guannan Ju [1,*], Lei Zhou [1], Chang Jiao [2], Jiafeng Shen [3], Yihao Luan [1,*] and Xinyu Zhao [1,*]

Citation: Ju, G.; Zhou, L.; Jiao, C.; Shen, J.; Luan, Y.; Zhao, X. One-Step Fabrication of a Functionally Integrated Device Based on Polydimethylsiloxane-Coated SiO_2 NPs for Efficient and Continuous Oil Absorption. *Materials* **2021**, *14*, 5998. https://doi.org/10.3390/ma14205998

Academic Editor: Won San Choi

Received: 16 September 2021
Accepted: 7 October 2021
Published: 12 October 2021

Publisher's Note: MDPI stays neutral with regard to jurisdictional claims in published maps and institutional affiliations.

Copyright: © 2021 by the authors. Licensee MDPI, Basel, Switzerland. This article is an open access article distributed under the terms and conditions of the Creative Commons Attribution (CC BY) license (http://creativecommons.org/licenses/by/4.0/).

1. Folding Process of the Functionally Integrated Device

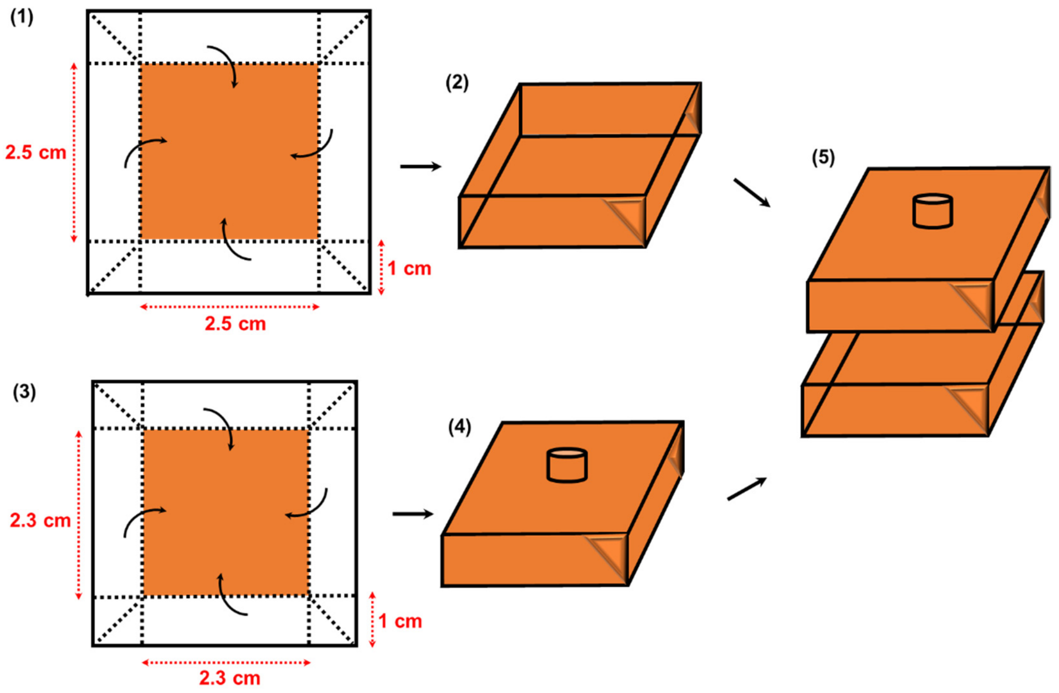

Figure S1. Illustration of the folding process of the functionally integrated device. Folding in the dotted line from the copper pieces ((**1**) and (**3**)) to two open boxes ((**2**) and (**4**)), followed by a simple assembly process (**5**).

2. TEM Images and Optical Photograph SiO_2 NPs Solution

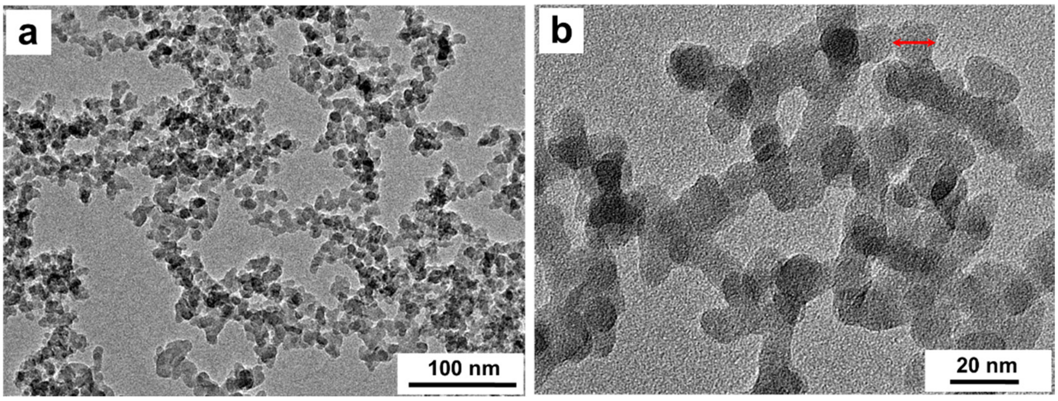

Figure S2. TEM images of SiO_2 NPs with (**a**) low magnification and (**b**) high magnification. Diameter of the as used SiO_2 NPs is about ~12 nm.

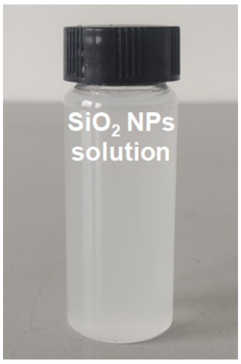

Figure S3. Ultrasonic dispersed SiO_2 NPs solution.

3. Influence of the Ratio of PDMS, Dilution Ratio and Spraying Time on WCA

As shown in Figure S4a, the as-prepared surface shows highest WCA of 152.1° when the ratio is 40%. This attributes to two reasons primarily: one is the suitable surface roughness of SiO_2 NPs stacking; another is low-surface-energy coating of PDMS. While the ratio is smaller than 30% or larger than 50%, the as-prepared surfaces do not show a satisfactory superhydrophobic property. This is mainly because insufficient PDMS would make SiO_2 NPs detach from the as-prepared surface due to lack of bonding, while excessive PDMS which forms a surface with less roughness. Therefore, we choose the optimal PDMS ratio of 40% as model system to fabricate the superhydrophobic surface. The dilution ratio of SiO_2 NPs and PDMS is important to form the superhydrophobic surface. From the Figure S4b, the WCA of as-prepared surface would decrease as dilution ratio increases. Obviously, the as-prepared surface loses superhydrophobicity (129.5°) when the ratio goes to 2.5. This is because the insufficient SiO_2 NPs cannot build the suitable surface roughness. As for dilution ratio from 0.5 to 1.5, the as-prepared surfaces still keep superhydrophobic property, although there is slight difference. Spraying time also affects the WCA of as-prepared surface. As shown in Figure S4c, as the spraying time increases from 10 to 80 s, the corresponding WCA gradually increases. This may be because the SiO_2 NPs stacking could form the dense micro-nano structures for building the superhydrophobic surface.

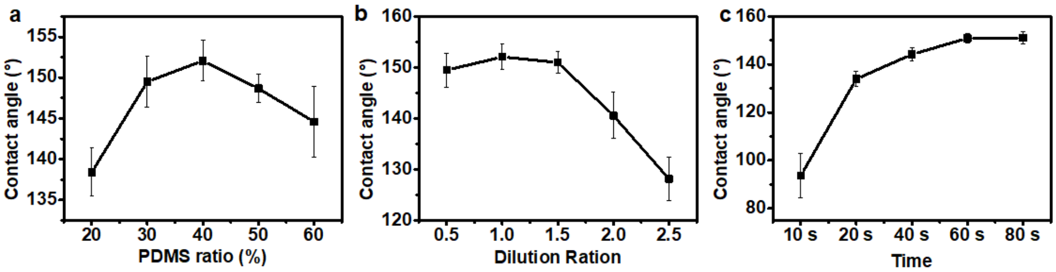

Figure S4. The effect of the ratio of PDMS (**a**), dilution ratio (**b**) and spraying time (**c**) on WCA.

4. SEM Images of Modification with Higher Magnification

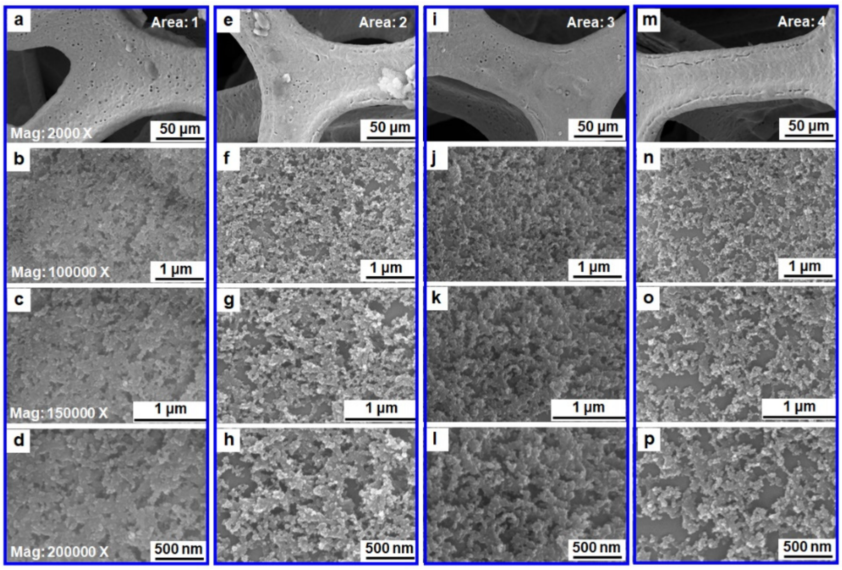

Figure S5. SEM images with different areas (area 1 (**a**–**d**), area 2 (**e**–**h**), area 3 (**i**–**l**), area 4 (**m**–**p**)) and magnification (2000×, 100000×, 150000×, 200000×) for modified copper foam.

5. Wettability Tests of as-Prepared Device

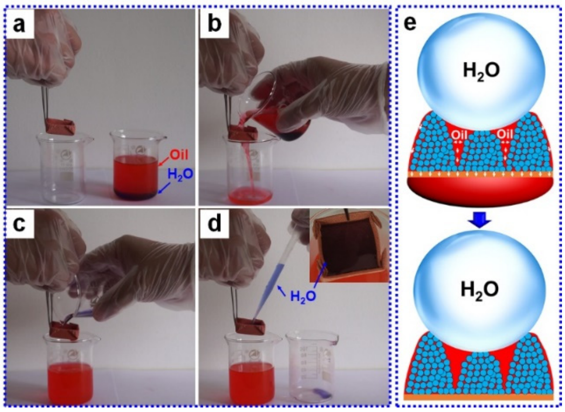

Figure S6. (**a**) The as-prepared device and the oil-water mixture. (**b**) The dyed-red n-heptane could be absorbed immediately and the excessive n-heptane could continuously penetrate the surface. (**c**, **d**) While the dyed-blue water would be blocked outside. (**e**) Schematic diagram of water and oil permeation process.

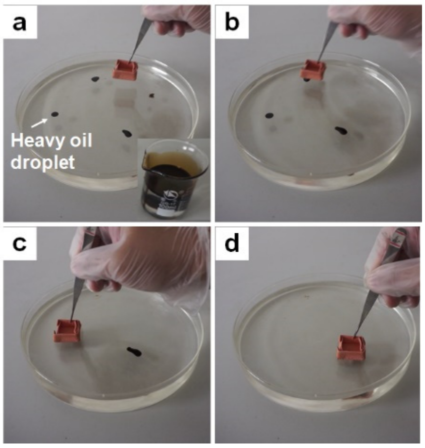

Figure S7. (**a**) Optical snapshots of oil droplets floated in water. (**b–d**) Removal of the oil droplets located on different sites by the as-prepared device.

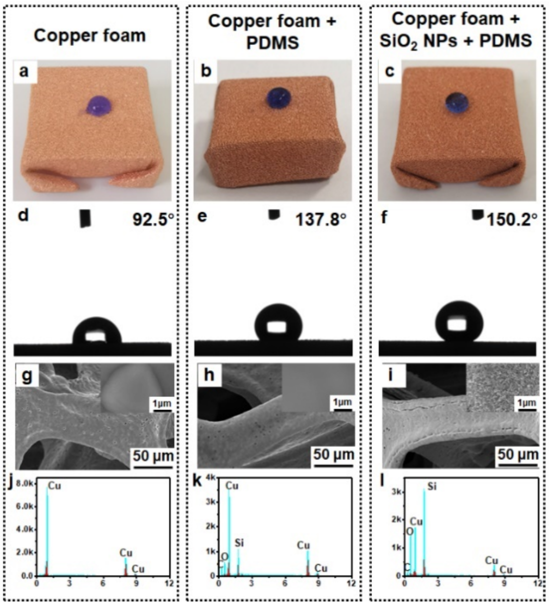

Figure S8. Photos of water droplets placed on the copper foam (**a**) before and (**b, c**) after the PDMS or PDMS/SiO_2 NPs modification. Corresponding WCA (**d–f**), SEM (**g–i**) and EDS (**j–l**) of as-prepared surface.

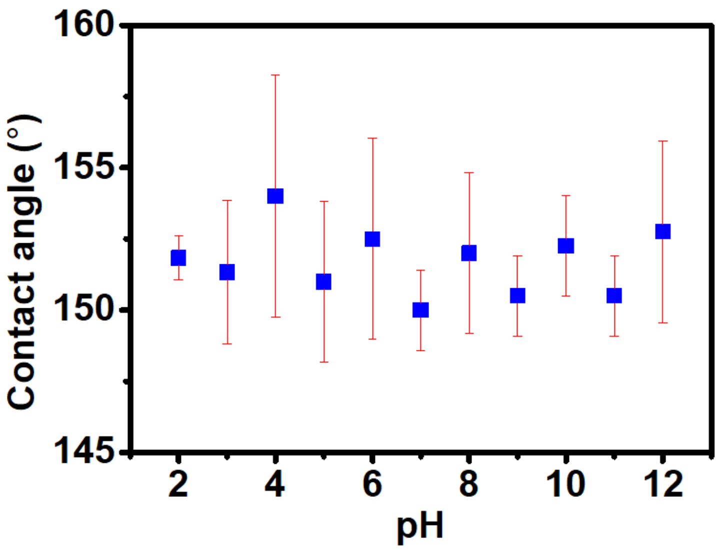

Figure S9. Variation of WCA of the as-prepared device under the different pH value.

6. The Evaluation of the Continuous Oil-Water Separation of the as-Prepared Device

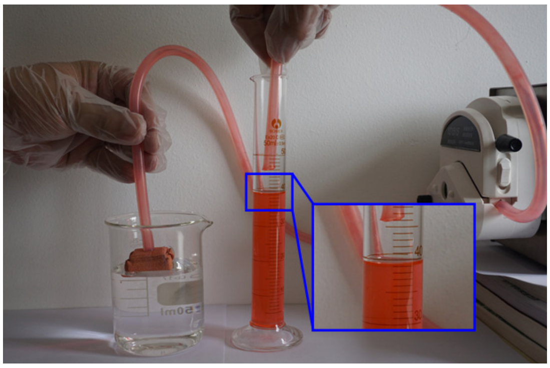

Figure S10. After the residual oil in the pump tube was poured out, the collected oil volume was about 39.2 mL.

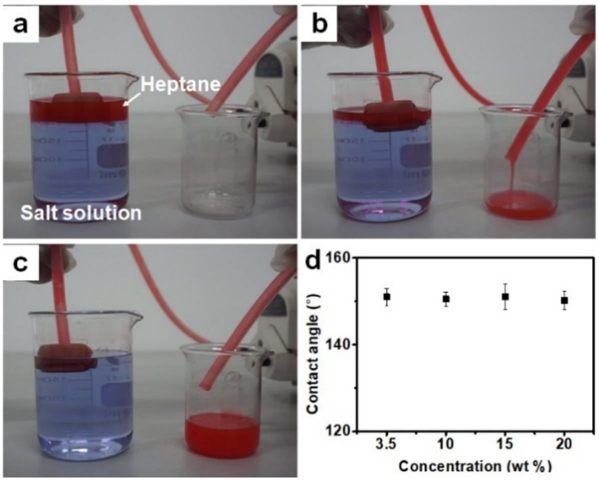

Figure S11. (**a**–**c**) Stepwise snapshots in the continuous oil-water separation and collection process in salt solution. (**d**) The WCA under different salt concentration.

Besides, our FID can also absorb oil underwater (Figure S12). By virtue of the pump, the simulated heavy oil underwater (i.e. dyed-red dichloromethane) is completed separated and collected from water.

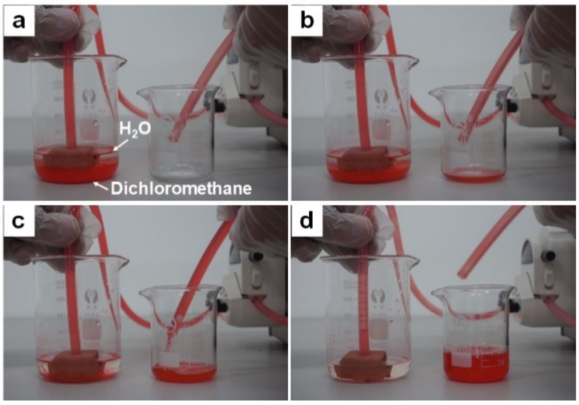

Figure S12. (**a**) Optical snapshots of the dichloromethane-water mixture. (**b–d**) The absorbing process under the water surface by the FID.

The reusability and stability of the as-prepared FID were investigated by testing the recycle number and separating efficiency. As shown in Figure S13, the as-prepared FID shows the high oil-water separating efficiency and high WCA after each cycle in the first four cycles, indicating the relatively high reusability and stability of the superhydrophobic FID. However, both the separating efficiency and WCA start to decrease after five cycles. This may be because that a small portion of SiO_2 NPs particles is peeled off from the FID surface after multiple washing-drying-testing cycles due to the relatively weak adhesion of SiO_2 NPs on the surface.

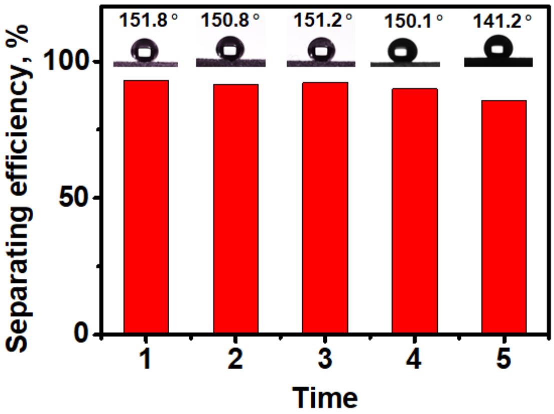

Figure S13. Recycled number and separating efficiency of the as-prepared FID in oil-water separation process. Inset: the corresponding WCA of the FID after each cycle.

Video S1. The oil-water separation process of the as-prepared device.

Video S2. The separation of salt oil-water mixture.

Video S3. The continuous and controllable oil-water separation with ON-OFF-ON function.

All the videos are accelerated to 3 times.
